# Case of Caries and Subsequent Removal of the Whole Inferior Maxilla

**Published:** 1871-10

**Authors:** R. H. Preston

**Affiliations:** Newboro'.


### SELECTED ARTICLES.

### ARTICLE V.

### *Case of Caries and Subsequent Removal of the Whole Inferior Maxilla.*

By R. H. Preston, M. D., Newboro'.

Sir,—Dr. Preston, at my request, sent me the accompanying particulars of his most extraordinary case, and I should have forwarded it to you sooner had I not wished to be able to report the condition of the subject of it, at a later date. I heard from him last week, and the report is that he has continued to improve slowly but steadily from the time of the removal of the bone, and that he considers himself perfectly recovered, the only thing preventing it being the inability of the dentist to find sufficient footing for a plate of teeth on the lower jaw. This was running in the man's mind from the first; for before proceeding to remove the jaw he was particularly anxious to know how soon after its removal he would be able to have a set of artificial teeth. I need hardly say that the reply was not very encouraging.

Yours, &c.,

Octavius Yates, M. D.

---

Mr. L. B., aged 46, a farmer residing in the Township of Bastard, Co. of Leeds, Ont., a man of spare but temperate habits was attacked on the 18th of October, last with severe pain in the second molar tooth, right side of the lower jaw. The tooth was decayed so as to expose the nerve. Pain was severe, and the face soon began to swell until the 4th day when suppuration ensued, but instead of finding relief his symptoms became more severe; the discharge increased, also the swelling which extended along the course of the bone. He went on in this way until the 28th Oct., when I was sent for. I found him laboring nnder high constitutional excitement, pulse running 150, skin hot and dry with pus discharging freely from around the decayed tooth.

With great difficulty I succeded in opening his mouth enough to extract the tooth and the one in front of it, both being quite loose. I ordered beef tea, chicken soup, egg, cream and brandy, to be given freely ; also put him on syrup of iodide of iron, and gave him a wash of carbolic water and glycerine.

On the 31st saw him again, the swelling and soreness greater and extending round the jaw ; pus was oozing from the side of every tooth on the right side. The constitutional symptoms more severe, hectic, night sweats, pulse 150, growing weaker and very drowsy. Beef tea, &c., continued, brandy increased.

Nov. 3rd, saw him again ; found him much weaker, disease extending, pus escaping from around every tooth in the whole jaw, and in large quantity ; removed more teeth ; increased as much as possible the amount of nutriment and stimulants.

Nov. 8th, saw him again and found him apparently sinking. The quantity of discharge was full a pint in 24 hours, a thick yellow-greenish pus, feet and legs œdematous, pulse weak and ranging from 130 to 150. At about this time, three weeks from the onset of the pain, besides continuing the nutriment &c., I gave him a large dose of quinine, also gave him cod liver oil. For the next three weeks I saw him twice a week—he lives over twenty miles from me or I should have visited him oftener—and during this time the discharge gradually became less, and he rallied in strength so that he was able to sit up for a short time every day.

Nov. 28th, Dr. Addison, of Farmersville, saw him with me, and we decided to remove all the teeth, hoping thereby o save the body of the bone, but soon after their removal —one was left— the gum fell from the bone. I then removed the greater portion of the alveola, when the condition of the body of the bone was discovered. The sloughing of the bone continued to go on rapidly. I then sent for Dr. Octavius Yates, of Kingston, who met me on the 24th Dec., when we removed the whole bone, cutting it in the mesial line and taking out first one and then the other side, and only requiring to use the handle of a scapel to separate the soft parts. No cutting was required, and only one or two teaspoonfuls of dark venous blood lost. By following with the finger, the track left by the boue, the glenoid cavity could be distincty felt, sound and free from disease. At the point of the chin a slight cartilaginous band could be felt, no doubt nature's commencing substitute for the jaw.

For some time there continued more or less œdema of the lower extremities, but it has now quite disappeared. The chin has contracted but very little while his cheeks are fuller than formerly, and although his voice is changed his articulation is perfectly distinct. His gums, or what is left of them, are gradually becoming harder, and he now eats hashes, puddings, &c., to such an extent that he weighs 15 pounds more than his usual weight before he became ill.

The bone itself, but for one sound tooth which remains, would, at first sight, hardly be recognized. The surface of the bone only here and there is preserved, while the whole interior portion seems to be lost. The bone or rather the pieces may be seen, having been added to the museum of the Royal College, Physicians and Surgeons, Kingston.

In conclusion, the question naturally arises, what was the cause of this rapid and complete destruction ? No constitutional, hereditary or acquired taint can be traced or found. No other part of the body was, or has yet, been affected. If left to itself—the supporting treatment excepted—the bone would probably have been thrown off or out, and thus furnish an ample spontaneous excision unheard of,—by me at all events—before meeting with this case.—*Canada Lancet.*

